# Perceptions and experiences of chair-based yoga by older adults with multimorbidity - a qualitative process evaluation of the Gentle Years Yoga randomised controlled trial

**DOI:** 10.1186/s12877-025-05782-3

**Published:** 2025-03-05

**Authors:** Lesley Ward, Garry A. Tew, Laura Wiley, Fiona Rose, Camila S. Maturana Palacios, Laura Bissell, Jenny Howsam, Tim Rapley

**Affiliations:** 1 Department of Social Work, Education and Community Wellbeing, Northumbria University, Newcastle uponTyne, UK; 2 Institute for Health and Care Improvement, York St John University, York, UK; 3 York Trials Unit, Department of Health Sciences, University of York, York, UK; 4 British Wheel of Yoga Qualifications, London, UK

**Keywords:** Older adults, Multimorbidity, Yoga, Healthy ageing, Biopsychosocial, Mental health, Sustainability, Process evaluation

## Abstract

**Background:**

Yoga is increasingly practised by older adults, with growing evidence for its safety and effectiveness across a range of health conditions common to the age group. This process evaluation, embedded within a randomised controlled trial of chair-based yoga for older adults with multimorbidity, qualitatively explored participants’ perceptions and experiences of the chair-based yoga programme.

**Methods:**

One-to-one interviews and class observations were conducted with a subset of trial participants randomised to receive the 12-week chair-based yoga programme. Interview participants were selectively recruited to represent the demographic breadth of the main trial cohort; one yoga class was observed at each delivery site. Interviews were audio recorded, independently transcribed, and analysed according to longitudinal and thematic analysis.

**Results:**

Twenty-five yoga participants were interviewed once (*N* = 10) or twice (*N* = 15), providing a 40-interview data set. Participants were aged 66–91 years (mean age 74 years), 56% female (*N* = 14), predominantly White British (*N* = 22, 88%), with 2–8 long term health conditions (mean 4.5 conditions). Four interlinked and overarching themes predominated: perceptions of healthy ageing, delineating yoga and exercise, yoga as an adaptable multifaceted health tool, and patterns of ongoing yoga practice. Participants equated acute symptom presentation, not multimorbidity, with illness, and mostly viewed their health as good. They distinguished yoga from exercise based on its integration of the breath with physical movements, which provided a mental focus unfound in other physical activities. Impact of the yoga programme ranged from minimal to transformative, dependent on meaningful biopsychosocial improvements. Accordingly, continuation of yoga beyond the trial ranged from none to full integration as a multifaceted health management tool.

**Conclusions:**

Participant experiences of the yoga programme interlinked views on health, ageing, exercise, and sustainable health management. Yoga presented as a safe, acceptable, and adaptable option for non-pharmacological health management in older adults. Impact on biopsychosocial health was variable, and directly linked to participants’ longer term yoga engagement. Education of health professionals and activity providers regarding ageist stereotypes of health and ageing, together with the evidence base for the safety and effectiveness of yoga, could support and broaden yoga’s reach and engagement among both older adult and multimorbid cohorts.

**Trial registration:**

ISRCTN ISRCTN13567538. Registered 18 March 2019.

## Introduction

The UK has an ageing demographic, with older adults [65 years and over] estimated at 20% of the population by 2028 [1]. Concomitant with this ageing society is an increased prevalence of multimorbidity, the presence of two or more long-term health conditions (LTHCs). Multimorbidity poses a substantial and increasing healthcare burden [2] and is now the norm rather than the exception among UK general practice consultations, independently associated with age, female gender, and deprivation [3]. Notably, a 34% prevalence of mental ill health in those with four or more physical conditions [4] highlights the need for a biopsychosocial management approach [5].

Recommendations for the management of multimorbidity increasingly promote personalised care, tailored to, and actively engaging, the individual [5, 6]. Recognising the potential risks of adverse drug reactions of multimorbidity-associated polypharmacy [7], guidance includes discussion of non-pharmacological treatment options such as exercise programmes [5].

Yoga, a mind-body practice typically comprising physical, breathing, and relaxation practices [8], presents as an acceptable physical activity for multimorbid older adults. Commonly associated with a younger, female, educated demographic [9], yoga practice is increasing among older adults, potentially reflective of adults bringing their pre-established yoga practice with them into later life [10]. Additionally, chair-based styles of yoga are increasing accessibility of this physically based practice for those with mobility issues precluding floor-based practice. Results from a UK survey suggest yoga practitioners attribute a positive impact of yoga on their biopsychosocial health and sleep, and have adopted healthier lifestyle behaviours associated with diet, smoking, and obesity compared to UK population norms [11].

There is increasing, robust, evidence for the safety [12] and effectiveness of yoga across a range of LTHCs common to older adults, including low back pain [13], cardiovascular disease [14], chronic obstructive pulmonary disease [15], osteoarthritis [16], anxiety [17], and cognitive function [18]. Given this evidence base, yoga is now a recommended practice across several clinical guidelines [19–21]. However, accessible chair-based forms of yoga specifically targeting multimorbid older adults is a relatively new area of research, and accordingly, trials to date are mostly of a small scale, and focussed on single health conditions within this cohort (e.g [22, 23]).

### Overview of the Gentle Years Yoga trial

The Gentle Years Yoga (GYY) trial sought to further the current evidence base of yoga for older adults, through a randomised controlled trial (RCT) with nested process evaluation, of a functionally inclusive, chair-based form of yoga for older adults with a range of physical and mental LTHCs. The full trial protocol is published elsewhere [24]. Briefly, community dwelling adults aged 65 years and over, with a minimum of two LTHCs, were recruited from 15 general practices across England and Wales (*N* = 454) and randomised to receive a 12-week chair-based yoga programme (*N* = 240) or usual care (*N* = 214). The yoga programme comprised weekly 75-minute group-based yoga classes with optional 15-minute post-class social time and supplementary home-practice guides. Yoga content included physical, breathing, and relaxation-based practices. The programme was delivered at 19 sites across England and Wales by GYY-qualified yoga teachers. Delivery was both face-to-face (seven sites) and online via Zoom [25] (12 sites), in response to changing Covid-19 social restrictions over the trial period. Effectiveness of the RCT, reported separately to this paper and in line with CONSORT guidelines, was evaluated across a range of self-reported health-related quality of life and wellbeing outcomes [26].

### Aim

The aim of this nested qualitative process evaluation was to explore the experiences and perceptions of a purposely selected sub-sample of participants receiving the 12-week GYY programme on their health, wellbeing, and ongoing health management. The full GYY project (including the RCT and nested qualitative process evaluation) was funded by the NIHR Health Technology Assessment Programme (reference 17/94/36), with ethical approval received from the National Research Ethics Committee North East – York (24/04/2019; 19/NE/0072). The GYY process evaluation was theoretically informed by Normalization Process Theory which encompasses the introduction, embedding, and routinisation of interventions [27, 28]. Conduct and reporting of this process evaluation aligns with robust qualitative methodology [29] and the COREQ guidelines for reporting qualitative interviews [30].

## Method

### Study design

This study is a qualitative process evaluation, specifically and solely targeting participants who received the 12-week GYY programme delivered in the intervention arm of the main trial. The study design included the collection of qualitative data from one-to-one interviews (single time point and longitudinal) and observations of yoga classes.

To ensure breadth of data, interviews were conducted at various timepoints across and beyond the 12-week programme, from October 2020 to May 2022. Additionally, a demographically representative sub-sample of the interview participants were invited back for a second (longitudinal) interview, to investigate the long-term sustainability of health impacts and yoga practice. To minimise positive selection bias, we invited participants who had indicated a range of experiences of the yoga intervention at their first interview. As per the initial interviews, sample size of the longitudinal interviews was determined by meaning saturation.

Interview schedules were semi-structured; core interview questions broadly explored participants’ general health and lifestyle, perceptions of yoga, biopsychosocial impact of the yoga programme, and future intentions for yoga. Follow-up interview schedules additionally explored any changes in a participant’s health status and yoga practice over time. Core questions were worded broadly and neutrally to minimise leading participants down a specific responce path, with prompts used to further explore their answers. Exemplar interview questions are provided in Table 1.

### Recruitment

The process evaluation purposely recruited from the cohort of 240 participants who had previously been randomised to receive the 12-week GYY programme. Participants who both indicated interest in the process evaluation interviews on their main RCT consent form and were subsequently randomised to the intervention arm to receive the 12-week yoga programme were eligible for this study. No further inclusion criteria were imposed.

Due to high interest in the process evaluation, not all eligible participants could be interviewed. Instead, a sampling strategy was designed to encompass both breadth and depth of data. Sample size was determined by meaning saturation of the interview data [31], and purposive sampling was iteratively conducted by experienced qualitative and process evaluation researchers (TR, LW). Sampling determinants focussed on risk factors for long term health conditions which overlapped with key demographics of yoga practitioners. These included gender, age, socioeconomic status (using the index of multiple deprivation [IMD] scores, an indicator of relative deprivation across the UK, as a proxy), and ethnicity. These determinants were then explored in relation to the number, type, and self-reported severity of a participant’s long term health conditions.

Identified participants were contacted via phone or email (LW) to discuss the interview process. If still interested, they were sent a process evaluation information pack and consent form; declining participation in the interviews did not impact participants’ main trial status. Once written informed consent was received, an interview time was arranged.

### Data collection

At the beginning of the process evaluation, the qualitative data collection was conducted face-to-face; interviews were conducted in participants’ homes and yoga classes were observed at the site of delivery. Following the implementation of Covid-19 restrictions, which restricted social gatherings and necessitated the move from face-to-face to online delivery of the GYY yoga classes, the process evaluation data collection also moved online. Interviews were conducted via telephone or Zoom, as per the participant’s preference, and the online yoga classes were observed via Zoom. Based on unanimously positive participant feedback regarding using Zoom and telephone for interviews, and ongoing Covid-19 concerns related to travel safety across the data collection period, remote collection of interview and class observation data continued for the remainder of the intervention period.

For both face-to-face and remote data collection, interviews were audio-recorded, independently transcribed, anonymised, and checked against the original audio file for accuracy. Interview data was supplemented with field notes from class observations; where possible, observations were conducted prior to an interview to enable depth of discussion around a participant’s yoga experience. All interviews, observations, and transcription accuracy were carried out by an experienced qualitative researcher (LW), with a sample of transcription further checked for accuracy by an experienced process evaluation researcher (TR).

### Analysis

Analysis of the qualitative process evaluation data was iterative throughout the study, and interview schedules adjusted in response to emergent data (TR, LW). Analysis was conducted in line with standard procedures of qualitative [29], longitudinal [32], and thematic [33] analysis. Firstly, individual pen portraits were written for each participant. Secondly, their data was transferred to an Excel spreadsheet and categorised into thematic groups. Thirdly, participants’ data was collated across groups and summarised. Interim findings were presented at the main GYY trial and data management meetings, enabling discussion and input from the wider trial team into the interview schedules and thematic groupings.

**Table 1:** Exemplar of core semi-structured interview questions

Question category	Sample question
About Your Health	What medical condition[s] concerns or impacts you the most, and why?
	What have you tried in the past to help manage your health/conditions?
Perceptions of yoga	What does ‘yoga’ mean to you - What did you think ‘yoga’ would be?
	Did you have any specific targeted reasons for wanting to try yoga?
Home practice	Which yoga practices do you choose to practice at home? Why/for what purpose?
	How do you integrate the home practice into your day?
Impact of yoga	How do you feel yoga has affected you?
	Have you experienced any untoward medical events as a result of the yoga?
Sustainability	What are your intentions for continuing with yoga or any other form of exercise/physical activity following the trial?
	If similar yoga classes were offered near to you, but not for free, how much would you be willing to pay to attend?
Specificity	Do you think older adults need classes specifically for them, or is it more about providing classes that are health appropriate?

## Results

### Demographics

Twenty-five yoga participants were interviewed once (*N* = 10) or twice (*N* = 15), providing a 40-interview data set. One participant declined a second interview, and one participant was unable to be contacted for follow-up. First interviews spanned the period from Week 4 of the 12-week intervention to 7 weeks post-intervention; duration 35–97 min. Follow-up interviews were conducted from 17 to 36 weeks post-intervention; duration 15 to 98 min.

Participant demographics encompassed the breadth of the intervention arm of the wider RCT cohort (Table 2): 56% female (*N* = 14), aged 66–91 years (mean age 74 years), predominantly White British (*N* = 22, 88%), and IMD scores ranging from 1 to 10. Sixteen participants lived with others, and nine lived alone (one in sheltered accommodation). Participants self-reported 27 types of physical and mental LTHCs (mean 4.5 conditions per participant, range 2–8), impacting movement, breathing, and social engagement.

**Table 2:** Characteristics of the 25 interview participants

Demographic	*N* (%)
Class delivery format	
Online	18 (72)
Face-to-face	7 (28)
Age (years) at Interview 1	
Mean (SD)	74 (8)
Range	66–91
Gender	
Female	14 (56)
Male	11 (44)
Ethnicity, n (%)	
White British	22 (88)
Other	3 (12)
Number of long term health conditions	
2	3 (13)
3–5	15 (60)
6 or more	7 (28)
Most common long term health conditions	
Arthritis (including osteoarthritis, rheumatoid arthritis, ankylosing spondylitis)	16 (64)
Bowel problems (including IBS, diverticulitis, inflammatory bowel disease)	9 (36)
Cardiovascular disease (including coronary heart disease, hypertension, heart failure, peripheral arterial disease)	14 (56)
Chronic back pain (including sciatica and spinal stenosis)	8 (32)
Depression or anxiety	8 (32)
Neurological conditions (including stroke, Parkinson’s Disease, cerebellar ataxia	3 (12)
Osteoporosis or osteopenia	4 (16)
Overweight or obesity	7 (28)
Sensory conditions (including hearing loss, macular degeneration, cataracts, glaucoma)	9 (36)
Index of multiple deprivation (IMD)	
1–4	6 (24)
5–7	5 (20)
8–10	14 (56)
Residential status	
Living alone in the Community	8 (32)
Living with other people in the Community	16 (64)
Living in sheltered housing with support	1 (4)

### Thematic outcomes

Participant feedback regarding the chair-based GYY programme was diverse, ranging from non-interest to transformative. Four interlinked and overarching themes predominated, regarding perceptions of healthy ageing, delineating yoga and exercise, yoga as an adaptable multifaceted health tool, and patterns of ongoing yoga practice. To add context to the accompanying quotes, participants age, gender, and number of LTHCs is provided.

### Health and ageing

For participants, it was important to first provide context around their health and ageing before addressing the role of yoga within that landscape. They collectively emphasised that multimorbidity should not, by default, be equated with ill health. Instead, most viewed their health as good, noting it was the acute presentation, not the chronic presence, of a health condition that determined its impact. Accordingly, multimorbidity was not presented as cumulative; due to surgical or pharmaceutical interventions reducing or eliminating symptoms, those with a higher number of health conditions did not necessarily describe themselves as experiencing poorer health than those with fewer conditions and vice versa. Furthermore, health status was not linearly attributed to life stage; several participants had experienced negative health since their early 30s, while others described good health and fitness into their 80s and 90s:

*‘I had a right hip replacement just two years ago*,* and that’s gone very well […] I’ve got high blood pressure too. That’s well controlled on medication. What else? I’ve been on antidepressants for about ten years now*,* and again that’s reasonably well controlled.’* Male; 68 years; 8 LTHCs.

Independent of health status, participants noted a negative societal attitude to ageing, with health professionals and media representations associating reduced health and fitness as an inevitable part of the ageing process. Conversely, participants viewed this concept of ‘older adults’ as outdated; several participants were employed full-time, had younger families, or active volunteer or carer roles. As such, many cited little in common with other participants, and felt a more representative grouping for the GYY trial classes would have been health status rather than age:

*‘I know we were all of a certain age but that was probably the only common denominator really. We all had different issues and problems*,* and we all lived differently*,* so I think that was the only thing was we were all of a certain age.’* Female; 66 years; 5 LTHCs.

Participants advocated a proactive approach to health management. However, whilst stating above that ageing did not preclude an active lifestyle, they added the caveat of physically based activities being age-appropriate and adaptable to the acute variability of conditions such as rheumatoid arthritis. High-intensity physical exertion was viewed as potentially harmful for certain health conditions, and a common reason for disinterest, or discontinuation, of gym-based exercise:

*‘Well an exercise class is aerobic. That means that you’re getting out of breath and sweaty. That’s not good for people in their nineties. So no*,* because it’s too much of a strain on the heart. You can’t do aerobics when you’re 90*,* because you’re straining your heart and your lungs and everything.’* Female; 90 years; 3 LTHCs.

### Yoga or exercise?

While acknowledging the intrinsic physical nature of yoga, participants varied in their consideration of whether yoga was a form of exercise. Most participants had neither tried nor considered yoga prior to the GYY trial but were familiar with its concept through practising family members, generational knowledge from its popularity during the 1960s, or media representation. Preconceptions of yoga included it being an Eastern spirituality practice, a gentle form of stretching, and a physically challenging exercise practised by fit younger adults, women, and the financially comfortable. All participants had previously thought yoga a mat-based practice, and most were unaware it could be adapted to either a seated practice or to older adults:

*‘I had no idea that yoga could be done specifically tailored to older people from a seated position. I viewed they were both a lot of standing*,* floor exercises.’* Female; 77 years; 2 LTHCs.

These preconceptions impacted participants’ views of the GYY classes. Those equating yoga with exercise found the classes physically unchallenging and questioned its potential benefit as a form of exercise. However, they saw the value of yoga as a safe and accessible gateway exercise for those coming from a sedentary lifestyle or taking up exercise for the first time in later life. What did challenge them were the integrated breathing practices:

*‘I didn’t feel it was at all physically challenging. What was challenging was the complexity of the movements*,* making certain you replicated what [trial yoga teacher] was doing*,* so there was a lot of thinking: “hang on*,* that’s out*,* then up and breathe out and breathe in”*,* you know [laughs].’* Male; 83 years; 3 LTHCs.

Coordinating their movements with their breathing was something they had not been taught, or experienced, in previous forms of exercise or rehabilitative exercise such as physiotherapy. And it was this unique feature of yoga which provided a mental focus not found in their other forms of physical activity. Conversely, participants interested in the broader non-physical aspects of yoga had little interest in practising yoga for exercise and clearly distinguished between the two practices. For them, exercise was an external, sweaty practice that you ‘do’, while yoga was an internalised, reflective practice that you ‘feel’:

*‘It was almost like exercising from the inside out. I just invented that phrase. I don’t know if you know what it means. But rather than sort of these big noisy things*,* you know*,* imposing on you*,* saying*,* “Do this*,*” it was… Well*,* I was being told to do it*,* but it still felt like a very personal*,* individual thing.’* Male; 68 years; 3 LTHCs.

This internalisation, achieved through integrating the physical, breathing, awareness, and philosophical aspects of yoga, enabled participants to work with themselves on a psychological level not found with exercise.

### Multifaceted health tool

Participants described their multimorbidity symptoms as an interconnected negative feedback loop involving pain, sleep disturbance, fatigue, mobility, and stress. As such, it was difficult to silo symptoms to a particular condition, or to know how and when to effectively self-manage their issues. The impact of the GYY classes on this feedback loop ranged from minimal to transformative. Those noting no health-related benefits from the 12-week yoga programme generally described a state of good health and physical activity when entering the trial or preferred a more physically challenging class targeting a specific health issue. Conversely, others cited biopsychosocial improvements during and beyond the 12-week intervention, using the multifaceted practices of yoga to address both acute health needs and ongoing functional maintenance.

### Physical impact

Self-reported physical benefits of the GYY classes included improved muscle strength, postural awareness, mobility, balance, coordination, and upper body range of motion, and decreased musculoskeletal pain and stiffness. Benefits were primarily associated with joint mobilisation and gentle range of motion movements. Participants noted these simple, ‘finer’ movements supplemented activities such as walking and gardening which targeted larger muscle groups.

Participants further associated postural, balance, and pain improvements with the mindful breathing practices of yoga. These techniques heightened spatial awareness with the external environment, leading to confidence in movement and reduced fear of falling both within and outside the home environment. Participants additionally used these techniques for acute pain management. Mindful breathing enabled them to detach from, and/or work through, their pain, in turn reducing its negative feedback on their sleep and mental health. These benefits, not associated with exercise, led to the unique experience of some participants being pain-free for the first time in years:

*‘‘Well*,* basically*,* I was fine for a short while*,* I had no pain at all. And that doesn’t happen to me [laughter]. That really doesn’t happen to me*,* I never have no pain. And I was amazed.’* Female; 68 years; 8 LTHCs.

### Psychosocial impact

For many participants, the most transformative benefits of the GYY classes were improved sleep, emotional, and mental wellbeing. These improvements were associated with the mindful breathing and relaxation practices of yoga, viewed by many as the defining feature between yoga and exercise. As with the physical practices, simplicity of the techniques was key, with many participants stating the basic act of becoming aware of their breathing was revelatory:

*‘It’s the simple things again. You’re aware – breathing in the air and it’s cold*,* or cooler*,* and it comes out warm. I mean*,* I’ve got to 70 years of age*,* I’ve never realised that before.’* Female; 70 years; 4 LTHCs.

Several participants who found the GYY classes physically unchallenging noted these psychosocial benefits were the reason for continuing with their practice. For them, breath awareness induced a feeling of calm and mental quietude not associated with their other forms of physical activity:

*‘I would say it’s probably more helpful for my mental wellbeing than my physical. That’s the way I look at it anyway. Because I think the sort of physical side of things*,* you know*,* my heart*,* I think I see more of the sort of dog walking and the cycling as being beneficial to that*,* whereas the yoga is more beneficial I think to my*,* you know*,* my anxiety*,* my worrying and that side of things.’* Male; 68 years; 4 LTHCs.

One of the most transformative impacts of yoga was the reversal of the negative feedback loop of stress, sleep disturbance, and fatigue. Again, breathing and awareness practices were used to subdue negative ruminations and induce physical and mental calm, and became a powerful tool for managing stress-related issues and improving sleep:

*‘I mean*,* we don’t get very much good news as it is*,* do we? And I can mull that over*,* which stops me sleeping. So what I tend to do is concentrate on the breathing of the yoga*,* and visualising*,* and then I can block out these other unhelpful thoughts*,* and in no time I can fall asleep.’* Female; 70 years; 4 LTHCs.

### Pharmaceutical impact

Participants expressed a common dislike of polypharmacy, with concerns over medication side effects and opiate dependency. Additionally, many viewed pharmaceuticals as simply masking symptoms, rather than improving health issues, and were interested in exploring new approaches to their health management.

Based on their experiences of the GYY trial yoga intervention, participants described practising or ‘*using’* yoga as both complementary and alternative to their pre-existing healthcare management. For example, yoga complemented what participants perceived as necessary disease-modifying medications for rheumatoid arthritis, by providing tools to manage associated sleep and fatigue issues which were either overlooked by health professionals or for which they were only offered pharmaceutical management options. Yoga also provided non-pharmacological options for acute-phase management, with several participants describing the effect of yoga practice as comparable to prescribed medications but with the benefit of no side effects:

*‘If you can get the same benefit*,* or almost the same benefit*,* and not take drugs that you don’t need*,* I think that’s a good idea.’* Female; 68 years; 8 LTHCs.

Yoga not only managed acute phase episodes, but often prevented trigger events such as stress-related rapid or shallow breathing from escalating into acute events such as anxiety or asthma attacks. As such, some participants described postural and breathing yoga techniques had replaced pharmaceuticals as their default form of acute symptom management for pain, anxiety, and asthma:

*‘I was getting stressed. And then I just sort of sat down and thought*,* “right*,* okay*,* sit down*,* take a breath”*,* and then breathe in through my nose*,* out through my nose*,* and just sat and relaxed instead of going for the Ventolin.’* Female; 70 years; 4 LTHCs.

### Patterns of ongoing yoga practice

Continuation of yoga beyond the GYY trial period ranged from none through to full integration into daily life. Discontinuation was associated with multiple factors. Some preferred more physically challenging exercise, while a lack of extrinsic motivation for self-practice was a barrier for others experiencing low mood or mental health challenges. Finally, a lack of meaningful benefit from, or interest in, yoga outweighed the response cost of adding a new activity to an already busy lifestyle:

*‘No*,* it’s not my thing*,* I must admit*,* at this moment. I think because*,* whatever benefit there is*,* I’ve not seen any big benefits which I can attribute to being yoga.’* Male; 66 years; 5 LTHCs.

Conversely, those with, or with the intention of, a sustained practice unanimously cited biopsychosocial benefits from the 12-week programme as their reason for continuation. For some, these benefits occurred at a level that meaningfully reduced the intensity and/or improved their ability to cope with their symptoms; for others, yoga provided a way to maintain their current level of health, mobility, and social connection. As such, yoga was as much about management of the person as it was management of the health condition:

*‘It’s not all about relieving the pain*,* but it’s like now – you know*,* it doesn’t*,* not all the time*,* it doesn’t actually make my condition better*,* but it makes me feel better. It makes me feel better.’ Female; 70 years; 4* LTHCs.

Sustainability of yoga practice was broader than the style and format of the classes the participants received in the trial. Through the 12-week programme participants gained a skillset enabling them to tailor the physical, breathing, and relaxation practices to their acute health needs, time commitments, and/or personal preferences. Thus, they described an evolving practice with three distinct paths. Firstly, some participants continued with instructor-led GYY classes, often preferring to continue with their online trial instructor rather than join a new, local GYY class. Secondly, some participants explored more challenging, mat-based styles or alternative forms of physical activity such as Tai Chi, which they viewed as more suitable to their physical ability. Thirdly, others found their newly acquired skillset provided the tools and knowledge to support ongoing independent practice, which could be integrated into their daily routine.

For many, the transformative impact of yoga placed an increased importance on their practice over time, with yoga becoming integral to their physical and mental health tool kit. Several participants with a sustained home practice began using yoga prescriptively, tailoring their daily practice to manage acute symptom presentation rather than following a generic pre-defined routine. Most commonly, joint mobilisations were used to address health and sedentary behaviour-related stiffness, and breathing techniques were used for managing acute symptoms of asthma and anxiety. Additionally, these participants used yoga ‘as needed’, due to its accessibility, invisibility, and immediate effectiveness:

*‘It calms me right away; I can literally*,* I can start it and within 10 seconds I’m into it. It’s so quick to do. So if you’re in a bad situation*,* like in the supermarket*,* I can even do it in the supermarket stood over the cabbages*,* if somebody’s being a dipstick around me.’* Female; 66 years; 5 LTHCs.

Independent of personal sustainability, all participants viewed yoga as a potentially safe and effective self-management tool for multimorbid older adults. Being handed self-responsibility for their practice empowered participants to manage their health in accordance with both their acute and chronic health needs. Additionally, for many with a background of physically intense exercise based on ‘no pain, no gain’, this gentler ethos of yoga proved transformative for viewing pre-held concepts of exercise from a new perspective:

*‘If it was a standing exercise*,* she’d say*,* “If this hurts*,* sit down and do it.” And so*,* I realised the point was not to make it hurt and end up having to take some ibuprofen*,* but just to gently stretch what you are able to do*,* and gradually you’re able to do more.’* Female; 77 years; 2 LTHCs.

Further supporting sustainability was participants’ opinion of yoga as compatible with the United Kingdom’s National Health Service remit of actively engaging individuals in their personal care. However, this came with the caveat that health professionals may need education on the evidence base of yoga for health management. Participants were particularly encouraging of the role of yoga for the management of mental health issues such as anxiety. Furthermore, they noted that as chronic health issues and acute injuries were independent of age, the target demographic for this chair-based style of yoga should be broadened to include health presentation in addition to age.

## Discussion

The aim of this process evaluation was to qualitatively investigate participant experiences and future potential of the GYY programme for the health and wellbeing of multimorbid older adults. Feedback suggested experiences were interlinked with participant views on health, ageing, and yoga’s relationship to exercise. The impact of yoga ranged from minimal to transformative, dependent on meaningful improvements in biopsychosocial health, and was directly linked to post-trial sustainability. Independent of personal experience, the GYY programme was viewed as a safe and acceptable health management option, with participants recommending the target demographic be redirected and broadened from age to health presentation.

Key to yoga’s suitability was its adaptability. Confirming the diversity of intrinsic capacity among older adults [34], participants emphasised their health and activity was not age-dependent and multimorbidity was not by default cumulative or interchangeable with illness. Instead, it was the acute, unmanageable presentation of a health condition which impacted day-to-day wellbeing and lifestyle, something many participants had experienced since young adulthood. Accordingly, health management needed to be adaptable to acute symptom variability and address concerns regarding dependency and adverse effects of pharmaceuticals [35]. Yoga, with its multi-faceted toolkit of physical, breathing, and awareness-based practices, provided some participants this adaptability and effectiveness, and presents as a favourable option for the non-pharmaceutical self-management of multimorbidity [5].

The self-reported physical and mental wellbeing benefits of the GYY programme for some reflect those of previous yoga interventions for older adults [36–38] and provide supporting evidence of older adults’ willingness to reduce polypharmacy [39, 40]. Mental health issues are highly prevalent in multimorbid populations [4] and increased among older adults in the wake of the Covid-19 pandemic [41]. Our findings suggest yoga provides an acceptable management option among these cohorts and supports previous findings of the importance of breathing practices for individuals with LTHCs [42]. Easily learned, able to be practised discreetly, and effective within several breaths, the simple practice of breathing with awareness became a management tool for acute asthma and stress-related episodes, replacing previous ‘as needed’ pharmaceutical management for some participants. Experiential feedback supports previous research associating yoga practices with emotional regulation and physiological markers of stress [43]. This is further supported by emerging research into the impact of yoga on the parasympathetic nervous system and gamma aminobutyric acid [GABA], and associated regulation of anxiety and stress [44, 45].

Not all participants reported benefit from the GYY programme, reflecting quantitative results from the GYY trial which indicated no significant benefits of the 12-week GYY programme compared to usual care across the primary and secondary outcomes [26, 46]. One potential reason may be suboptimal dosage to induce physiological or psychological change. However, the intervention dosage of 15 h was comparable with the common dosage of 13.3–16 h for chair-based yoga interventions for older adults [47]. A more likely explanation may be found in this qualitative data, whereby several participants indicated having comparatively good health and fitness at baseline, and those looking to GYY yoga as a new exercise option experiencing the GYY chair-based style physically unchallenging compared to their established exercise and physical activities.

Participant feedback generally reflected the concepts of active ageing and healthy ageing, which promote the role of behavioural determinants such as physical activity to manage functional health and enhance quality of life [34, 48]. Although these concepts are not new, evidence suggests ageist stereotypes about physical activity and exercise risks are compromising older adults’ leisure and exercise patterns [49–51]. While physically unchallenging for the more functionally independent participants, findings suggest the GYY programme provides a potential gateway into physical activity for those new or averse to exercise, or with a health condition precluding engagement in physical activity. Given the high levels of sedentary behaviour among older adults [52], and its association as a risk factor for LTHCs [53], chair-based yoga may play a role in initiating activity-based behaviour for the management of multimorbidity [5].

### Future implementation

While the quantitative results of the GYY randomised controlled trial, reported separately, indicated no overall benefit of the GYY yoga intervention on health-related outcomes compared to usual care [26, 46], in-depth feedback from this qualitative process evaluation of intervention participants suggests its potential as a health behaviour option. Broadening the GYY remit to include people based on their physical ability rather than age could provide accessibility to a younger demographic with chronic health needs, to individuals in transition from injury or surgery, and to experienced yoga practitioners no longer able to meet the physical requirements of a general yoga class due to onset or progression of health issues. Additionally, the GYY style of yoga may serve as a gateway into more physical floor-based styles for those who enjoyed the wellbeing impacts of the yoga breathing practices but found the chair-based physical postures unchallenging.

Promotion of yoga as a health management tool needs to address negative stereotyping of ageing and physical exercise by activity providers [51] and older adults themselves, transforming the conversation into one addressing the role of physical activity for active ageing [50]. Furthermore, education of health providers as to the safety and effectiveness of yoga is key to successful implementation. Despite non-pharmaceutical management of LTHCs being entrenched in health and clinical guidelines [5, 19–21], a lack of knowledge of the evidence base is a barrier for UK health practitioners recommending yoga to their patients [54], reflecting international trends in the link between knowledge and recommendation of complementary therapy use among health care professionals [55].

### Strengths and limitations

The robust methodology of this process evaluation aimed to minimise the inherent interviewer, self-selection, and sampling biases of qualitative methodology, which may unintentionally impart a positive experience bias to data collection. Minimising these three biases was achieved through methodological processes such as participants providing initial consent to the interviews prior to knowing which study arm they would be randomised to, and regular objective input from the wider trial team on any potential selection and interview bias by the interviewer. Additionally, the purposive choice of sampling determinants covering demographic factors and health presentation ensured representation of the interview participants reflected that of the wider trial demographic. Finally, iterative analysis of ethnographic and interview data ensured the interview schedules were congruent with new and developing participant experiences across the data collection phase.

A further, and unanticipated, strength of the process evaluation was the switch to telephone and online interviews in response to Covid-19 social distancing and travel restrictions which impacted face-to-face contact. Participant feedback indicated this interview format was preferable for many of them, due to variables including anxiety around meeting face-to-face with a researcher, lack of privacy to meet face-to-face, or busy schedules that better fitted around a phone call.

The fact that most participants presented proactive views towards yoga and positive lifestyle behaviours in their interviews is perhaps unsurprising given their chosen engagement in a yoga trial. However, purposive sampling for the follow-up interviews ensured representation of a broad range of trial experiences, with the semi-structured interview approach further enabling an exploration of participants’ underlying views of both their enjoyment or disinterest in yoga based on their trial experience.

While generalisation of qualitative data from the process evaluation interviews is limited by the small sample size relevant to the full trial cohort, the participants themselves provided reasoning for broadening the use of the study results beyond the GYY trial demographic. Based on this, the next step in this research process would be to explore the opinions of this recommended broader demographic in trying chair-based yoga as a tool for their health management. Additionally, of interest are the views of those older adults who declined trial participation, to explore the factors involved in their decision making.

## Conclusion

Participant experiences of the GYY programme, comprising chair-based yoga for older adults with multimorbidity, interlinked views on health, ageing, exercise, and sustainable health management. The impact of yoga ranged from minimal to transformative, dependent on meaningful improvements in biopsychosocial health, and was directly linked to post-trial sustainability. Independent of personal experience, participants viewed the GYY programme as a safe and acceptable option for non-pharmacological management of physical and mental health issues. Key to its suitability was the adaptability of the yoga practices to the variable acute presentation of health issues. As many participants’ health conditions had presented prior to the age of 65 years, they recommended the target demographic for the GYY style of yoga be refocussed on health presentation rather than age, to include younger adults living with long-term health conditions. Education of health professionals and activity providers could further support and broaden the potential reach of the programme, by addressing age-related stereotypes of health and ageing and highlighting the evidence base for the safety and effectiveness of yoga across a range of LTHCs.

## Data Availability

All relevant data are included in the manuscript.

## Abbreviations

GYY: Gentle Years Yoga
LTHC: Long term health condition
NIHR: National Institute for Health and Care Research
UK: United Kingdom
